# Publisher Correction: RAPP-containing arrest peptides induce translational stalling by short circuiting the ribosomal peptidyltransferase activity

**DOI:** 10.1038/s41467-024-47508-w

**Published:** 2024-04-15

**Authors:** Martino Morici, Sara Gabrielli, Keigo Fujiwara, Helge Paternoga, Bertrand Beckert, Lars V. Bock, Shinobu Chiba, Daniel N. Wilson

**Affiliations:** 1 Institute for Biochemistry and Molecular Biology, University of Hamburg, Martin-Luther-King-Platz 6, 20146 Hamburg, Germany; 2 Theoretical and Computational Biophysics Department, Max Planck Institute for Multidisciplinary Sciences, Göttingen, Germany; 3 Faculty of Life Sciences and Institute for Protein Dynamics, Kyoto Sangyo University, Kamigamo, Motoyama, Kita-ku, Kyoto, 603-8555 Japan

**Keywords:** Cryoelectron microscopy, Ribosome

Correction to: *Nature Communications* 10.1038/s41467-024-46761-3, published online 19 March 2024

The original version of this Article contained an error in Ref. 21, which was incorrectly given with the wrong publication date. The correct form of Ref. 21 is: Gersteuer, F., Morici, M., Gabrielli, S. et al. The SecM arrest peptide traps a pre-peptide bond formation state of the ribosome. *Nat Commun* **15**, 2431 (2024). This has been corrected in the PDF and HTML versions of the Article.

