# Supplementary material for: RAPP-containing arrest peptides induce translational stalling by short circuiting the ribosomal peptidyltransferase activity
Source: Nat Commun. 2024 Mar 19;15:2432. doi: 10.1038/s41467-024-46761-3 (PMC10951233; doi:10.1038/s41467-024-46761-3)
Supplement: Supplementary file 1 — Supplementary Information [file 41467_2024_46761_MOESM1_ESM.pdf]

Supplementary Information for

RAPP-containing arrest peptides induce translational stalling by short circuiting the ribosomal peptidyltransferase activity

Martino Morici[1], Sara Gabrielli[2], Keigo Fujiwara[3], Helge Paternoga[1], Bertrand Beckert[1], Lars V. Bock[2], Shinobu Chiba[3,*], Daniel N. Wilson[1,*]

[1] Institute for Biochemistry and Molecular Biology, University of Hamburg, Martin-Luther-King-Platz 6, 20146 Hamburg, Germany.

[2] Theoretical and Computational Biophysics Department, Max Planck Institute for Multidisciplinary Sciences, Göttingen, Germany

[3] Faculty of Life Sciences, Kyoto Sangyo University, Kamigamo, Motoyama, Kita-ku, Kyoto 603-8555, Japan.

*Correspondence to:

Daniel Wilson (Daniel.Wilson@chemie.uni-hamburg.de)

Shinobu Chiba (schiba@cc.kyoto-su.ac.jp)

Content:

Supplementary Figures 1-13

Supplementary Tables 1-5

Supplementary References

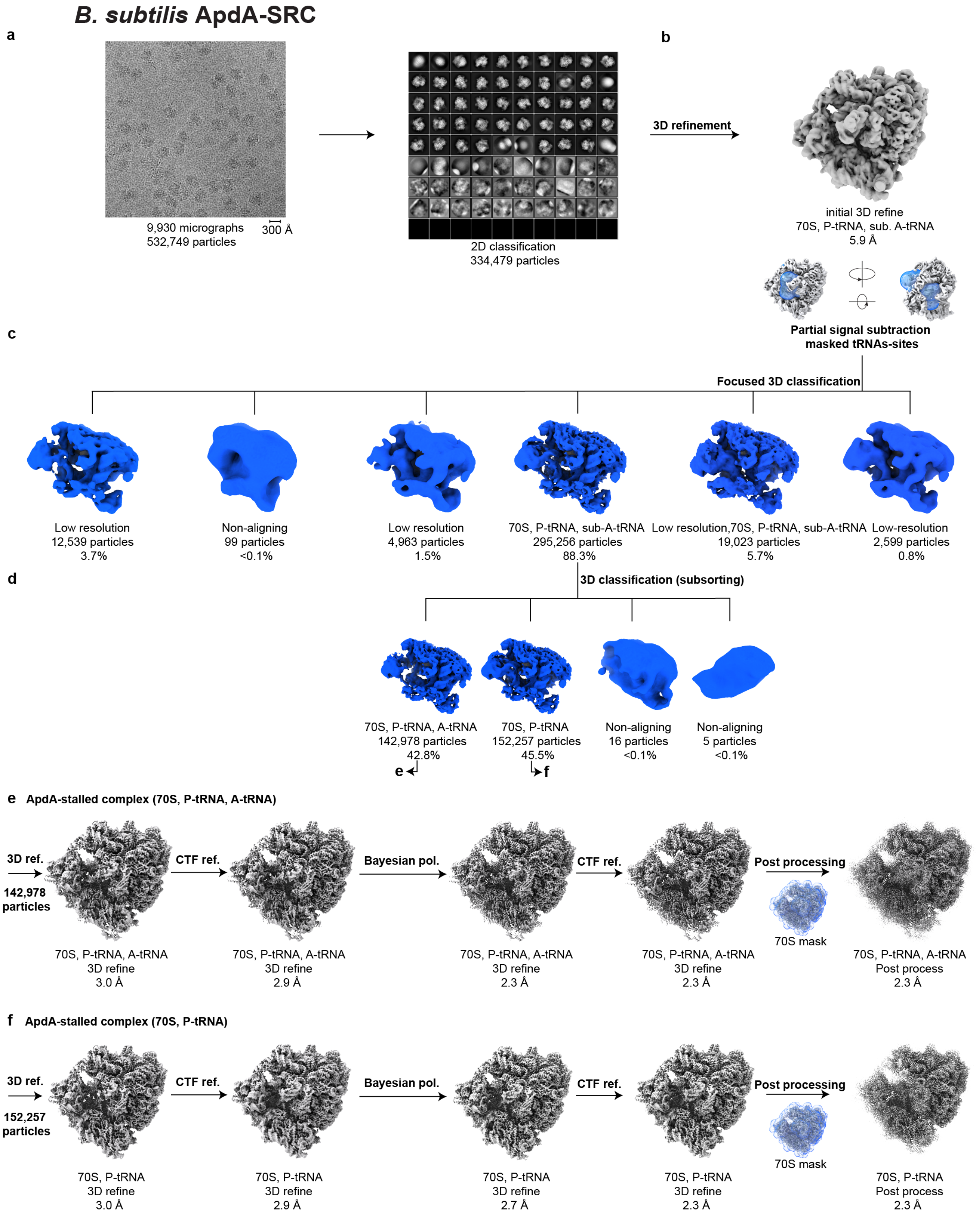

Supplementary Fig. 1 *In silico* sorting scheme for ApdA. (**a**) From 9,930 micrographs, 532,749 particles were picked and subjected to 2D classification resulting in 334,479 ribosome-like particles. Particles were (**b**) initially 3D-refined, then (**c**) subsorted into six classes using a mask around the tRNA binding sites. (**d**) The major class (88.3%) displayed P-tRNA and substoichiometric A-tRNA and was subsorted into four classes. (**e-f**) The two major classes with (**e**) both A- and P-tRNA density (42.8%), and (**f**) only P-tRNA (45.5%), were processed further, resulting in final average resolutions (at FSC 0.143) of 2.3 Å and 2.3 Å, respectively.

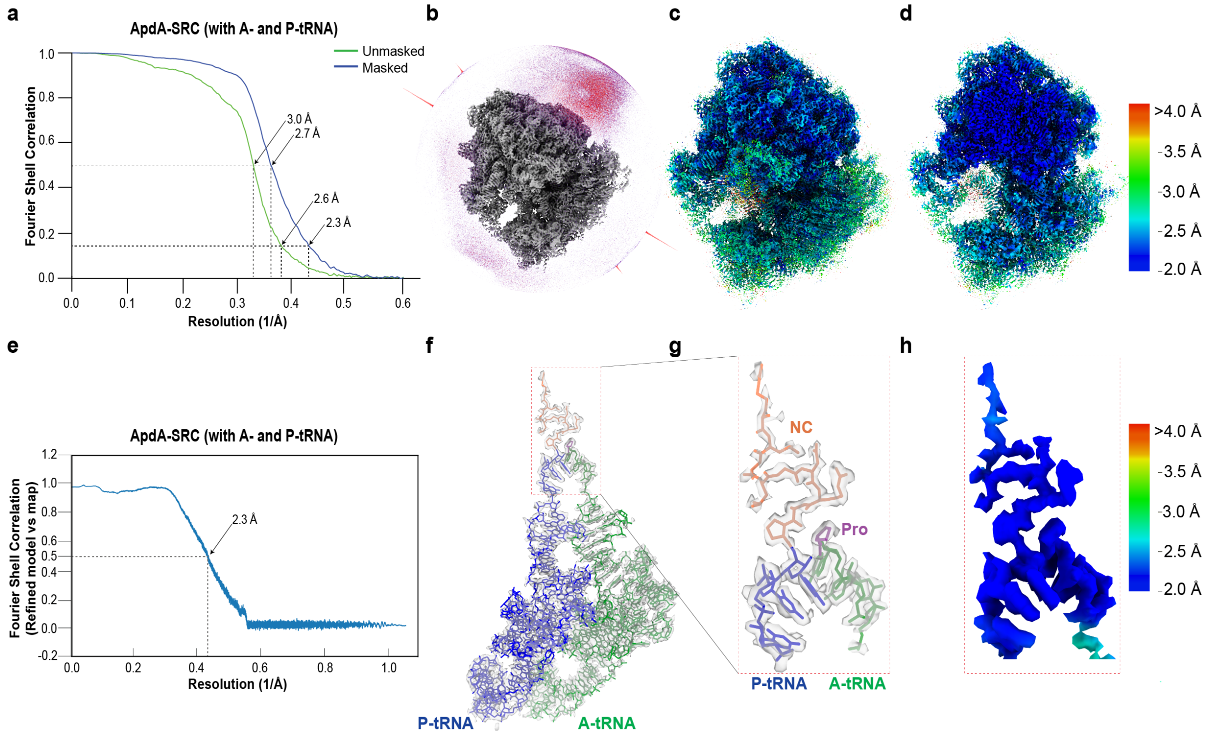

Supplementary Figure 2 FSC and local resolution for ApdA-SRCs. (**a**) Fourier shell correlation (FSC) curve of the ApdA-SRC containing A- and P-tRNA, with unmasked (green) and masked (blue) FSC curves plotted against the resolution (1/Å). (**b-d**) Cryo-EM density for the map of the ApdA-SRC, coloured (**b**) grey, and (**c-d**) according to local resolution. In (**b**), the Euler angular distribution of particles for the ApdA-SRC is shown with the height of the peaks representing the number of particles. In (**d**), a transverse section reveals the core of the 50S subunit, including the ribosomal exit tunnel. (**e**) Refined model vs map FSC curve of the ApdA-SRC containing A- and P-tRNA plotted against the resolution (1/Å). (**f-h**) Cryo-EM density (grey mesh) for the peptidyl-tRNA in the P-site and Pro-tRNA in the A-site of the post-processed map of the ApdA-SRC, coloured grey (**f,g**) and according to local resolution (**h**). In (f-g), the model for the A- and P-tRNA are shown in green and blue, respectively, with the ApdA NC in orange and the Pro in purple.

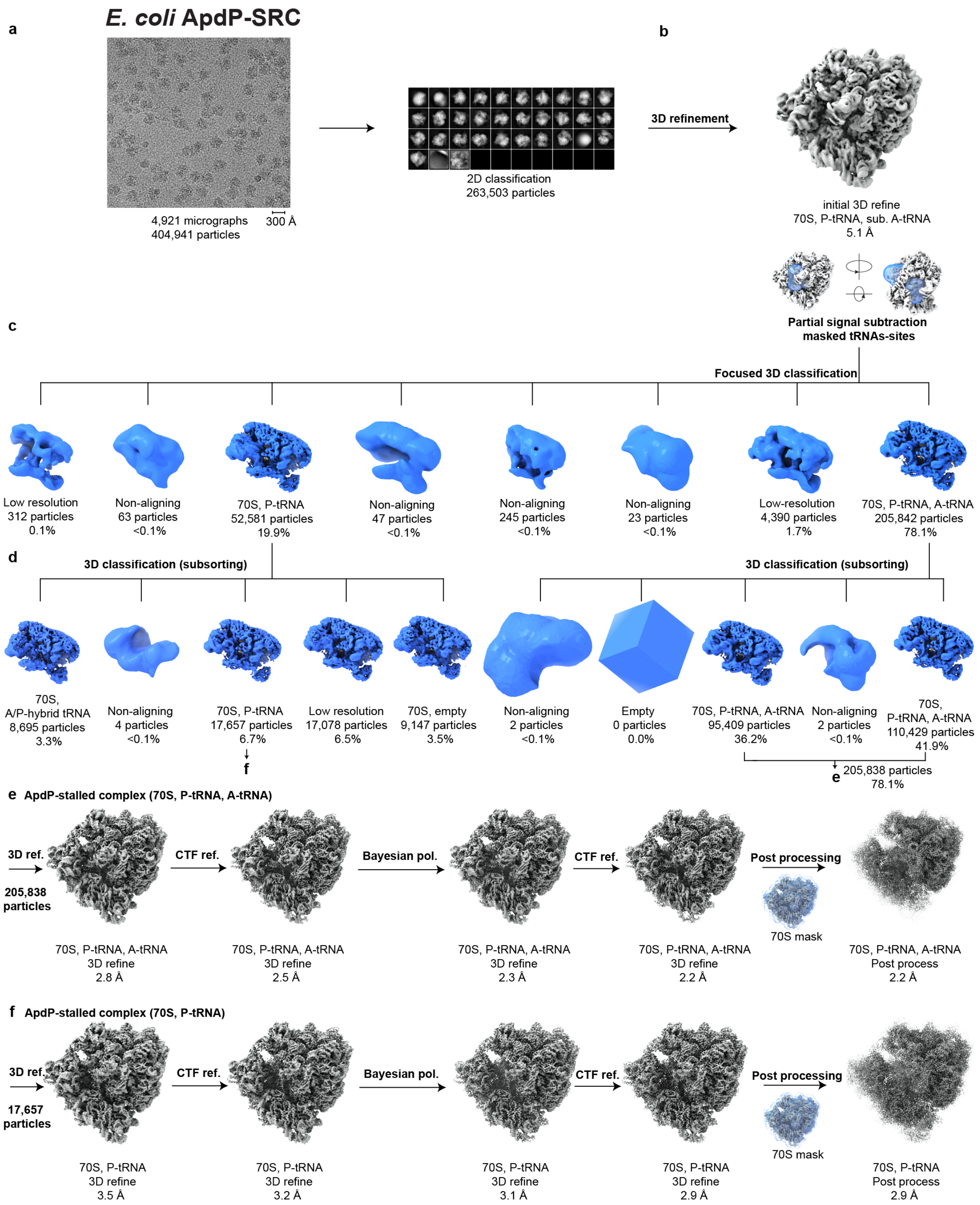

Supplementary Figure 3 ***In silico*** **sorting scheme for ApdP.** (**a**) From 4,921 micrographs, 404,941 particles were picked and subjected to 2D classification resulting in 263,503 ribosome-like particles. Particles were (**b**) initially 3D-refined, then (**c**) subsorted into eight classes using a mask around the tRNA binding sites. (**d**) The major class (78.1%) contained density for A- and P-tRNA and was further subsorted into five classes, two of which contained stoichiometric density for A- and P-tRNA and were therefore re-combined. A second major class (19.9%) from the initial sorting displayed density for the P-tRNA and was also subsorted into five further classes, one of which contained stoichiometric P-tRNA (6.7%). (**e-f**) The two major classes with (**e**) both A- and P-tRNA density (78.1%), and (**f**) only P-tRNA (6.7%), were processed further, resulting in final average resolutions (at FSC 0.143) of 2.2 Å and 2.9 Å, respectively.

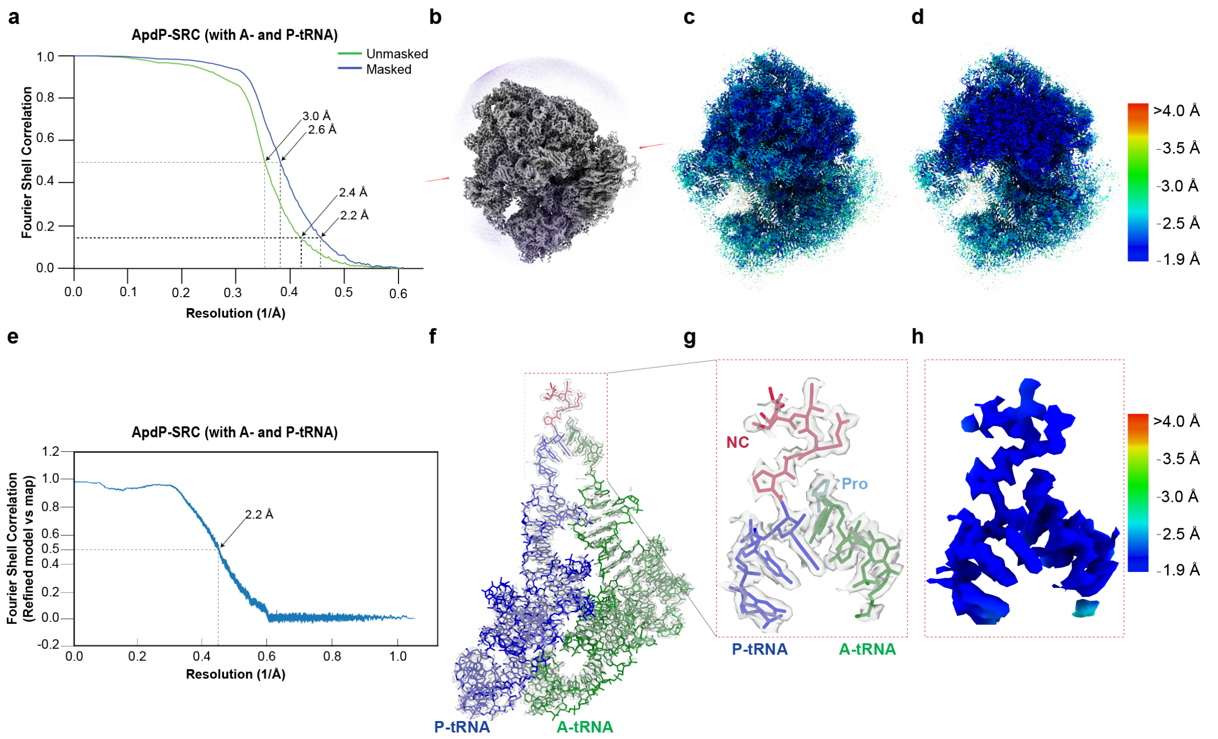

Supplementary Figure 4 FSC and local resolution for ApdP-SRC. (**a**) Fourier shell correlation (FSC) curve of the ApdP-SRC containing A- and P-tRNA, with unmasked (green) and masked (blue) FSC curves plotted against the resolution (1/Å). (**b-d**) Cryo-EM density for the ApdP-SRC map, coloured (**b**) grey, and (**c-d**) according to local resolution. In (**b**), the Euler angular distribution of particles for the ApdP-SRC is shown with the height of the peaks representing the number of particles. In (**d**), a transverse section reveals the core of the 50S subunit, including the ribosomal exit tunnel. (**e**) Refined model vs map FSC curve of the ApdP-SRC containing A- and P-tRNA plotted against the resolution (1/Å). (**f-h**) Cryo-EM density (grey mesh) for the peptidyl-tRNA in the P-site and Pro-tRNA in the A-site of the post-processed map of the ApdP-SRC, coloured grey (**f,g**) and according to local resolution (**h**). In (f-g), the model for the A- and P-tRNA are shown in green and blue, respectively, with the ApdP NC in red and the Pro in cyan.

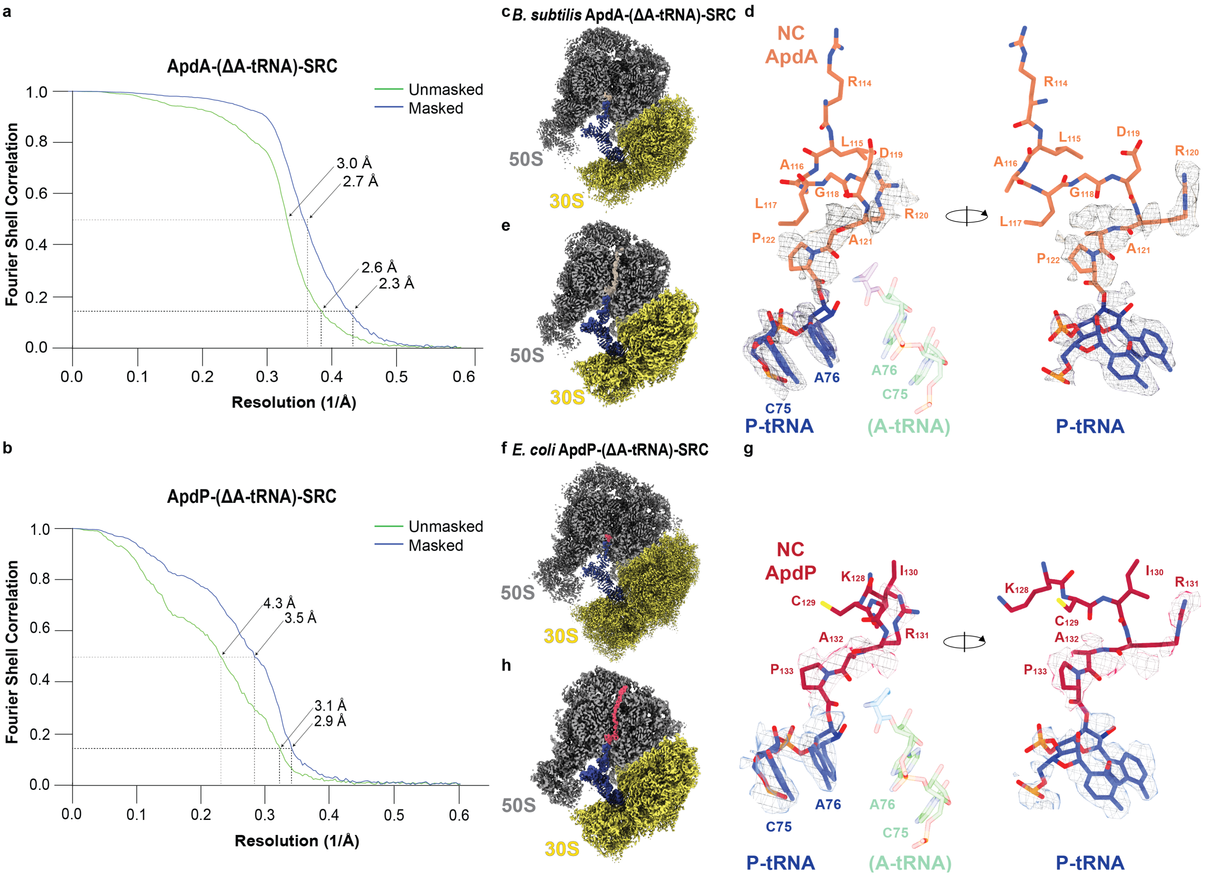

Supplementary Figure 5 FSC and cryo-EM density for ApdA- and ApdP-SRC without A-site tRNA. (**a-b**) Fourier shell correlation (FSC) curves of the (**a**) ApdA-SRC and (**b**) ApdP-SRC containing P-tRNA only, with unmasked (green) and masked (blue) FSC curves plotted against the resolution (1/Å). (**c**) Cryo-EM map of the post-processed *B. subtilis* ApdA-SRC lacking A-tRNA with transverse section of the 50S (grey) to reveal density for the nascent chain (tan), P-tRNA (blue), and 30S (yellow). (**d**) Two views showing the cryo-EM map density for the P-site tRNA of the post-processed *B. subtilis* ApdA-SRC. The P-site tRNA (blue) bears the ApdA nascent chain (orange) and the location of A-tRNA (green) is shown for reference. (**e**) as (**c**), but cryo-EM map of 3D-refined *B. subtilis* ApdA-SRC lacking A-tRNA. (**f**) Cryo-EM map of the post-processed *E. coli* ApdP-SRC with transverse section of the 50S (grey) to reveal density for the nascent chain (red), P-tRNA (blue), A-tRNA (green); 30S (yellow). (**g**) Two views showing cryo-EM map density for A- and P-site tRNAs of the post-processed *E. coli* ApdP-SRC. The P-site tRNA (blue) bears the ApdP nascent chain (red), and the location of A-tRNA is shown for reference. (**h**) as (**f**), but cryo-EM map of 3D-refined *E. coli* ApdP-SRC.

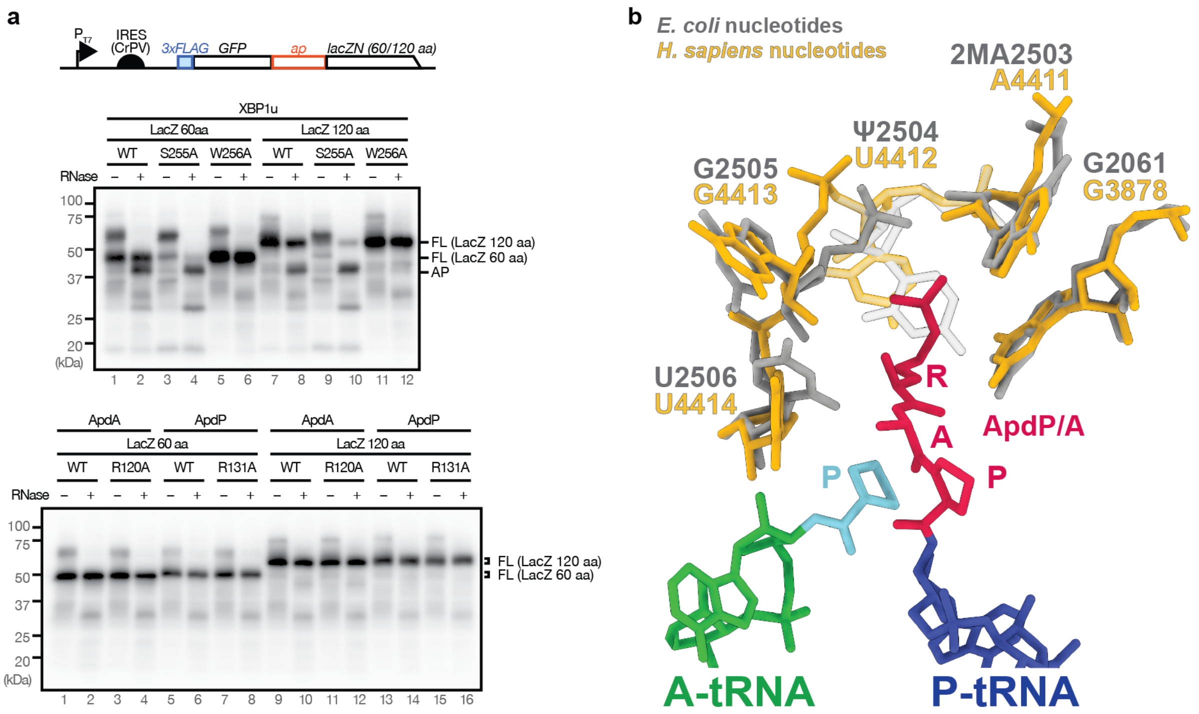

Supplementary Figure 6 Translation stalling of the RAPP motif in eukaryotic systems. (**a**) The coding region for wild-type (WT) or mutant derivatives of XBP1u(186–261), ApdA(39–128), or ApdP (34–140) was sandwich-fused between FLAG-*gfp* and either *lacZ60* or *lacZ120*, which encodes the N-terminal 60 or 120 residues of LacZ, respectively. The *in vitro* translation in the rabbit reticulocyte lysate was carried out at 30°C for 20 minutes. The translation products were analyzed by anti-FLAG immunoblotting. FL and AP represent the full-length and arrest species without tRNA moiety, respectively. The S225A mutation enhances the arrest of XBP1u, whereas the W256A mutation compromises the arrest. Experiments were performed in two independent experiments with similar results. Source data are provided in Supplementary Figure 13. (**b**) Overlay of the model of ApdP with 23S rRNA (grey) compared to the model of *Homo sapiens* translating ribosome (PDB ID 6olg)[1] showing the corresponding nucleotides of the 28S rRNA (gold). Alignment was performed between the 23S rRNA of ApdP and the 28S rRNA of PDB ID 6olg based on nucleotides of the PTC. *E. coli* numbering is used for ApdP, *Homo sapiens* numbering is used for PDB ID 6olg.

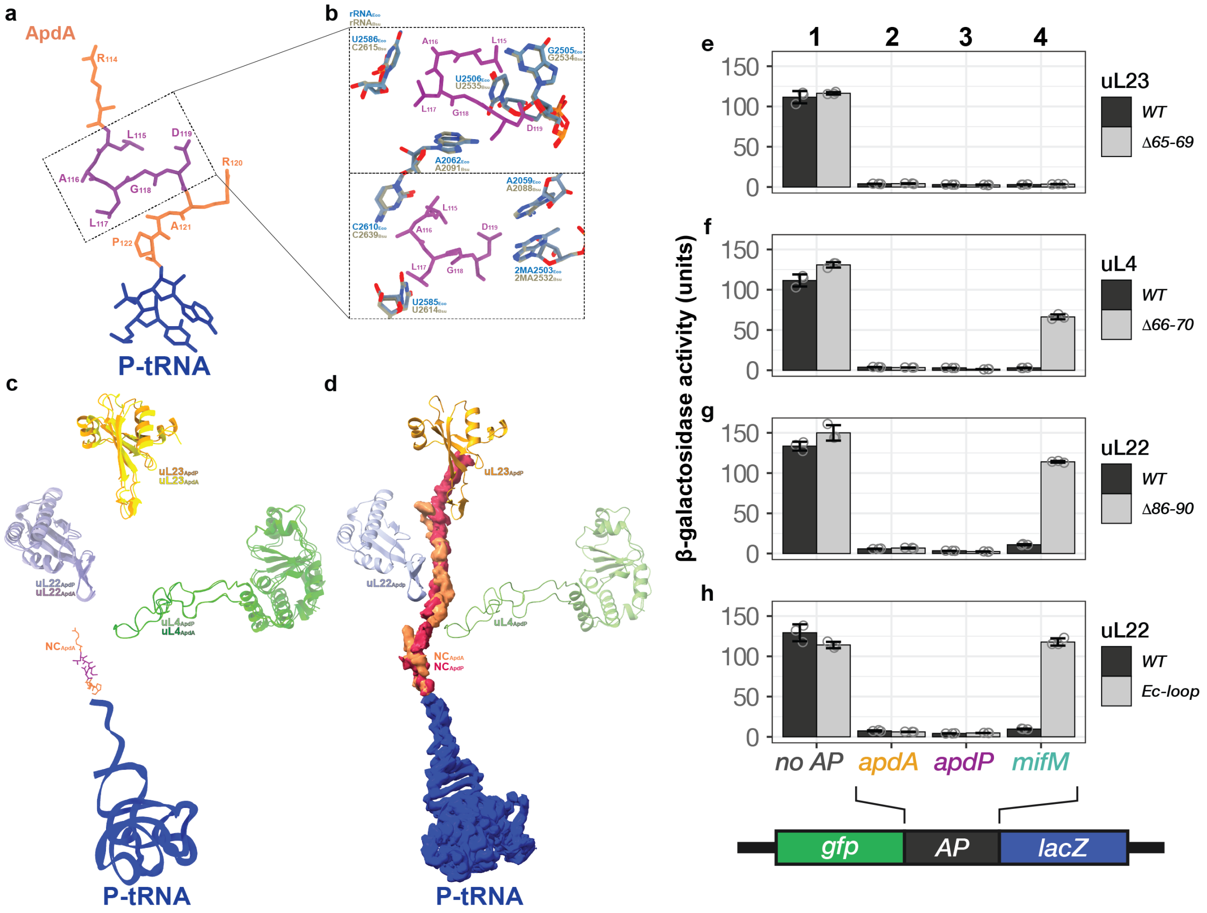

Supplementary Figure 7 Species-specificity of ApdA and ApdP stalling. (**a**) Structure of ApdA (orange) with residues (115-119) colored purple that enable stalling in *E. coli* when substituted with ApdP. (**b**) Superimposition of *B. subtilis* ApdA-SRC with *E. coli* 23S rRNA (from ApdP-SRC), revealing high conservation between the 23S rRNA nucleotides surrounding ApdA residues 115-119. (**c**) Relative proximity of residues 115-119 of ApdA to ribosomal proteins uL4 (green), uL22 (cyan) and uL23 (yellow). (**d**) Superimposition of filtered cryo-EM densities (based on the local resolution) for the ApdA (orange) and ApdP (red) nascent chains from 3D-refined maps of the ApdA-SRC and ApdP-SRC, revealing overall similarity in the paths of the nascent chains through the tunnel, however, slight differences in the vicinity of ribosomal proteins uL4 (green), uL22 (cyan) and uL23 (yellow). (**e-h**) Effect of the mutations in the ribosomal proteins in the nascent polypeptide exit tunnel on the translation arrest of ApdA, ApdP and MifM. β-galactosidase activity (mean ± s. d., N = 3) of the *lacZ*-based arrest reporter strains expressing wildtype (WT: black) and (**e**) uL23-Δ65-69, (**f**) uL4-Δ66-70, (**g**) uL22-Δ86-89 deletion mutants, and (**h**) Ec-loop (grey) variants of uL22, respectively. The *gfp-lacZ* without the coding sequence for the arrest peptide (AP) serves as a control (no AP). Each reporter gene is integrated into the *amyE* locus of the chromosome of the *B. subtilis* strains expressing wildtype or mutant derivative of uL4, uL22, or uL23 ribosomal protein for β-galactosidase assay. Source data are provided as a Source Data file

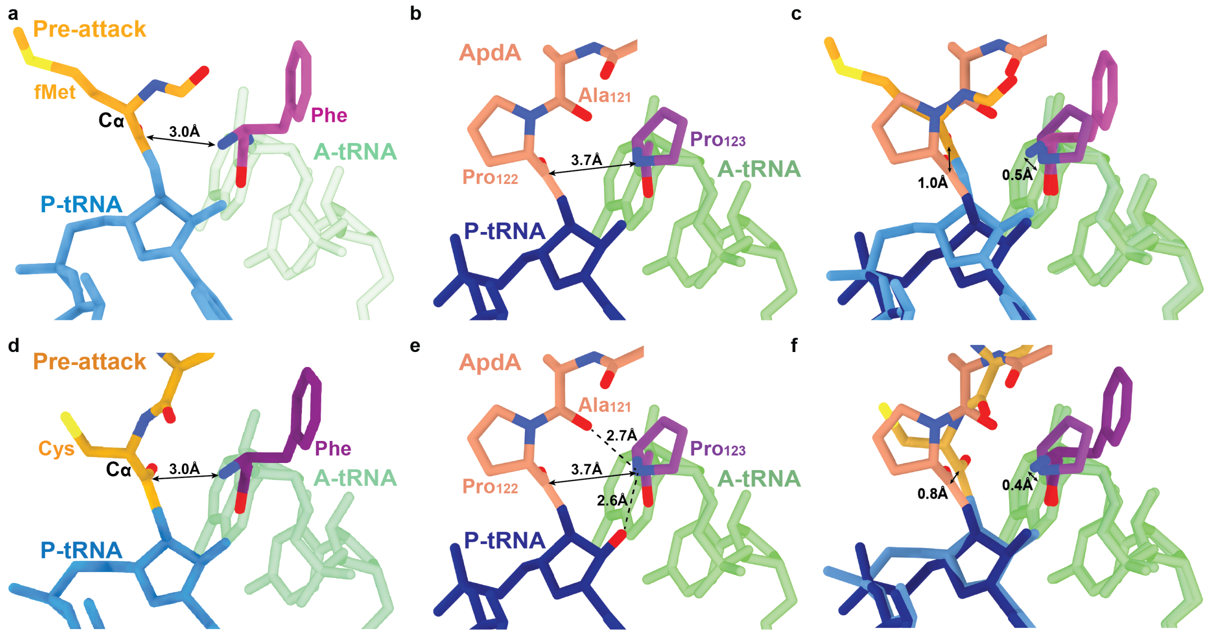

Supplementary Figure 8 Model for ApdA/ApdP-mediated translational stalling. (**a**) View of the PTC of a pre-attack state (PDB ID 1VY4)[2], showing a fMet-NH-tRNA (gold/blue) at the P-site and a phenylyl-NH-tRNA (purple/green) at the A-site. The distance (3.0 Å) between the attacking amine of the A-tRNA and the carbonyl carbon of the P-tRNA is arrowed. (**b**) Same view as (**a**), but for the ApdA-SRC with ApdA-tRNA (orange/dark blue) in the P-site and Pro-tRNA (purple/green) in the A-site. (**c**) Overlay of (**a**) and (**b**) (aligned on the basis of the 23S rRNA) highlighting the difference in the distance between the attacking amino groups at the A-site and the carbonyl carbons at the P-site. **(d)** View of the PTC of a pre-attack state (PDB ID 8CVK)[3], showing a tripeptidyl-NH-tRNA (gold/blue) at the P-site and a phenylyl-NH-tRNA (purple/green) at the A-site. The distance (3.0 Å) between the attacking amine of the A-tRNA and the carbonyl carbon of the P-tRNA is arrowed. (**e**) Same view as (**d**), but for the ApdA-SRC with ApdA-tRNA (orange/dark blue) in the P-site and Pro-tRNA (purple/green) in the A-site. (**f**) Overlay of (**d**) and (**e**) (aligned on the basis of the 23S rRNA) highlighting the difference in the distance between the attacking amino groups at the A-site and the carbonyl carbons at the P-site.

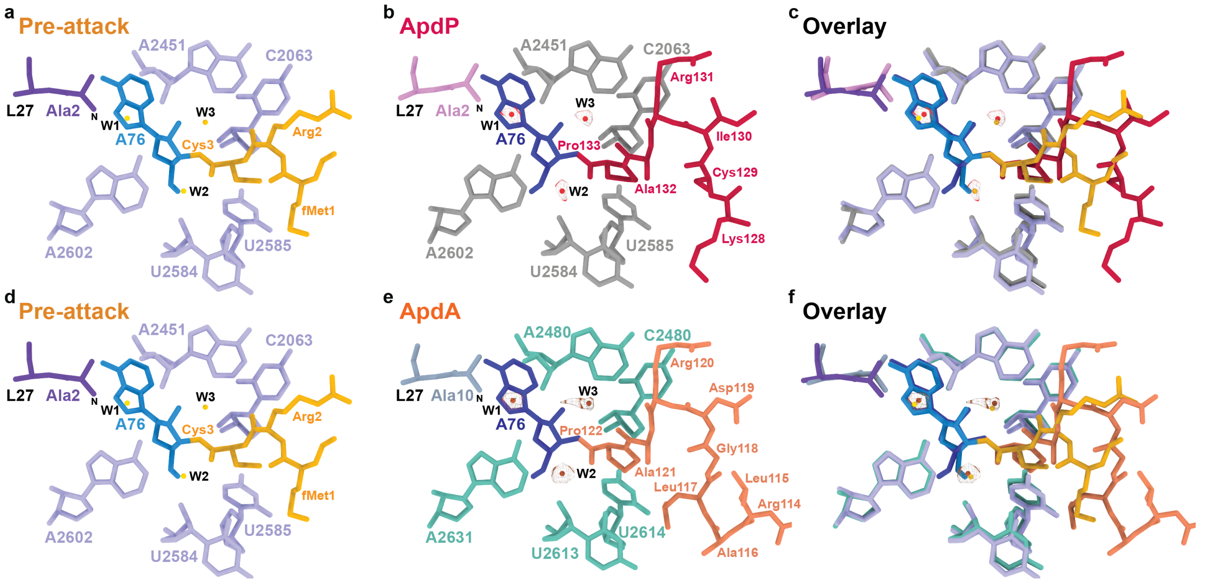

Supplementary Figure 9 Mechanism of stalling of ApdA and ApdP. (**a-c**) Components of the proton wire required for nucleophilic attack and peptide bond formation that are located around the nascent chain (gold/dark red) connected to A76 of the P-tRNA (light blue/dark blue), including nucleotides of the 23S rRNA (lilac/grey), the N-terminus of L27 (purple/pink) and waters (yellow/red with meshed density) from (**a**) a pre-attack state (PDB ID 8CVK)[3]and (**b**) the ApdP-SRC. The A-tRNA is omitted for clarity. *E. coli* numbering is used. (**c**) Overlay of (**a**) and (**b**) (alignment is based on the 23S rRNA). (**d-f**) Components of the proton wire required for nucleophilic attack and peptide bond formation that are located around the nascent chain (gold/orange) connected to A76 of the P-tRNA (light blue/dark blue), including nucleotides of the 23S rRNA (lilac/teal), the N-terminus of L27 (purple/silver) and waters (yellow/dark orange with meshed density) from (**d**) a pre-attack state (PDB ID 8CVK)[3] and (**e**) ApdA. The A-tRNA is omitted for clarity. *E. coli* numbering is used in panel (**d**), *B. subtilis* numbering is used in panel (**e**). (**f**) Overlay of (**d**) and (**e**) (alignment is based on the 23S rRNA).

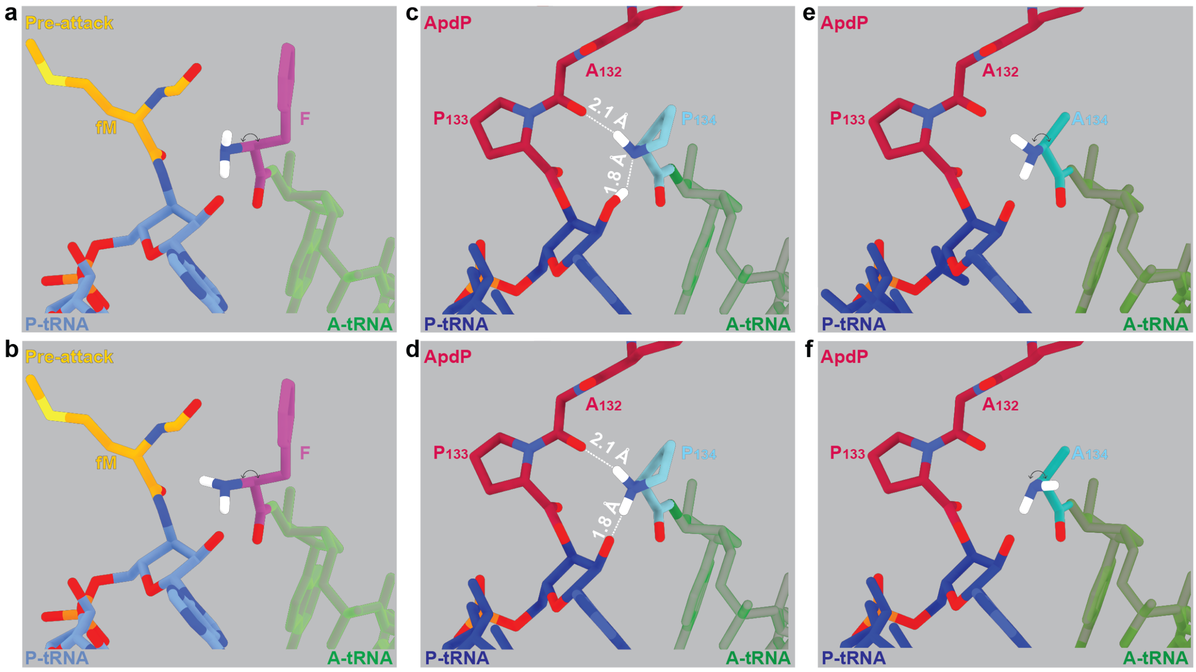

Supplementary Figure 10 Model for ApdA/ApdP-mediated translational stalling. (**a-f**) Focus on the PTC of a pre-attack (PDB ID 1VY4)[2] (**a-b**) coloured as Fig. 5a, with hydrogens coloured white, with the two main rotation configurations of the bond connecting the nitrogen and the α-carbon, of ApdP (**c-d**) coloured as Fig. 2e, with hydrogens coloured in white, with P_{134} in the unprotonated (**c**) and in the protonated form (**d**), and for the *in silico* mutation P134A (**e-f**) with the two main rotation configurations of the bond connecting the nitrogen and the α-carbon.

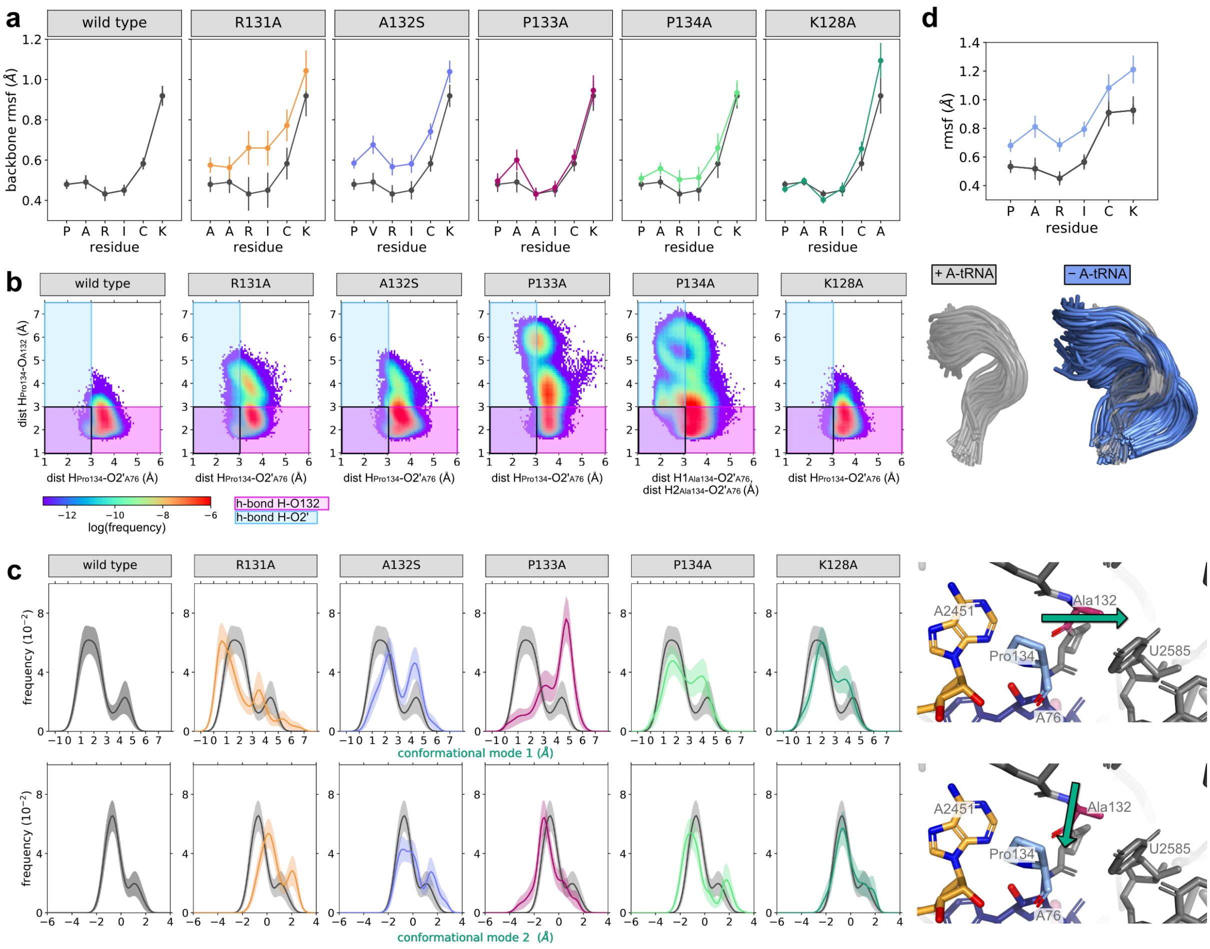

Supplementary Fig. 11 Effect of mutations and absence of A-tRNA on the flexibility of the ApdP peptide. (**a**) Comparison between the flexibility of wild type (grey) and variants (coloured). Flexibility is quantified by the root mean square fluctuation (rmsf) of the backbone atoms of each peptide residue. Mean and standard deviations from 20 independent simulations (**b**) 2d-histograms of the N-H(Pro134)-O(Ala132) vs N-H(Pro134)-2'O(A76) distances sampled by all conformations that also adopted N-H(Pro134)-C(Pro133) distances lower than 3.8 Å. The regions where H(Pro134)-2'O(A76) and N-H(Pro134)-O(Ala132) hydrogen bonds form, are highlighted in blue and pink, respectively. In all variants, the N-H(Pro134)-O(Ala132) bond forms more frequently than N-H(Pro134)-2'O(A76). However, compared to wild type and control, the non-stalling mutations allow for a more frequent disengagement of N-H(Pro134) from O(Ala132). Most of the conformations where the N-H(Pro134)-2'O(A76) hydrogen bond is formed, also display N-H(Pro134)-O(Ala132) distances larger than 3 Å. In wild type and control, instead, most of the conformations compatible with N-H(Pro134)-2'O(A76) hydrogen bond formation, are also compatible with the N-H(Pro134)-O(Ala132) hydrogen bond. (**c**) Left: distributions of the conformations sampled by the Ala132 backbone atoms along their first (top row) and second (bottom row) most dominant conformational modes. Lines and error bars were obtained from mean and standard deviation of 1000 bootstraps of 20 independent simulations. Right: the directions of the first (top row) and second (bottom) conformational modes are displayed by green arrows. The cryo-EM model of Ala132 and of its surrounding residues is shown as a reference (**d**) Top row: rmsf of the heavy atoms of each peptide residue in the presence (grey) and absence of the A-tRNA (blue). Bottom row: Visualization of the structural ensembles in the presence (grey) and absence of the tRNA (blue). Source data for the figure can be obtained from Zenodo (10.5281/zenodo.10426362).

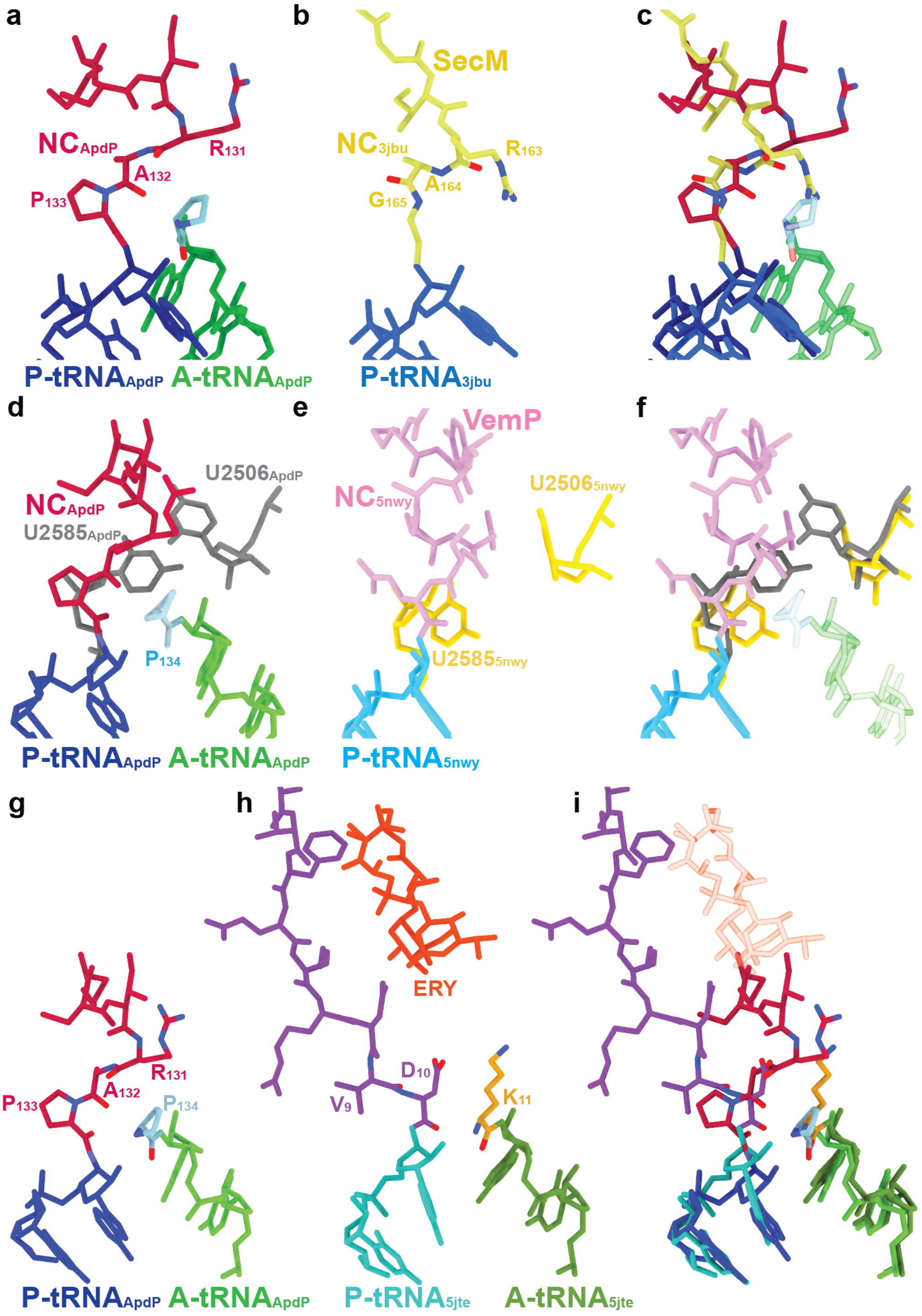

Supplementary Figure 12 Comparison of ApdP with SecM, VemP and ErmBL arrest peptides. (**a-c**) Same view of (**a**) ApdP (coloured as in Fig. 3b) and (**b**) SecM(PDB ID 3JBU)[4], and (**c**) overlay of (**a**) and (**b**). SecM nascent chain is coloured in yellow, its P-tRNA is light blue. The amino acid residues of the (**a**) RAP and (**b**) RAG motifs are coloured by heteroatoms. Alignment is based on the 23S rRNA. (**d-f**) Same view of (**d**) ApdP (A-tRNA is green, the prolyl residue P134 is cyan and the nucleotides are grey) and (**e**) VemP (PDB ID 5NWY)[5], and (**f**) overlay of (**d**) and (**e**). VemP nascent chain is coloured in pink, its P-tRNA is light blue and the nucleotides are yellow. *E. coli* numbering is used for this panel. Alignment is based on the 23S rRNA. (**g-i**) Same view of (**g**) ApdP (A-tRNA is green, the prolyl residue P134 is cyan and the nucleotides are grey) and (**h**) ErmBL (PDB ID 5JTE)[6], and (**i**) overlay of (**h**) and (**g**). ErmBL nascent chain is coloured in purple, P-tRNA (turquoise), A-site Lys-tRNA (green, with K11 orange) and erythromycin (ERY, orange).

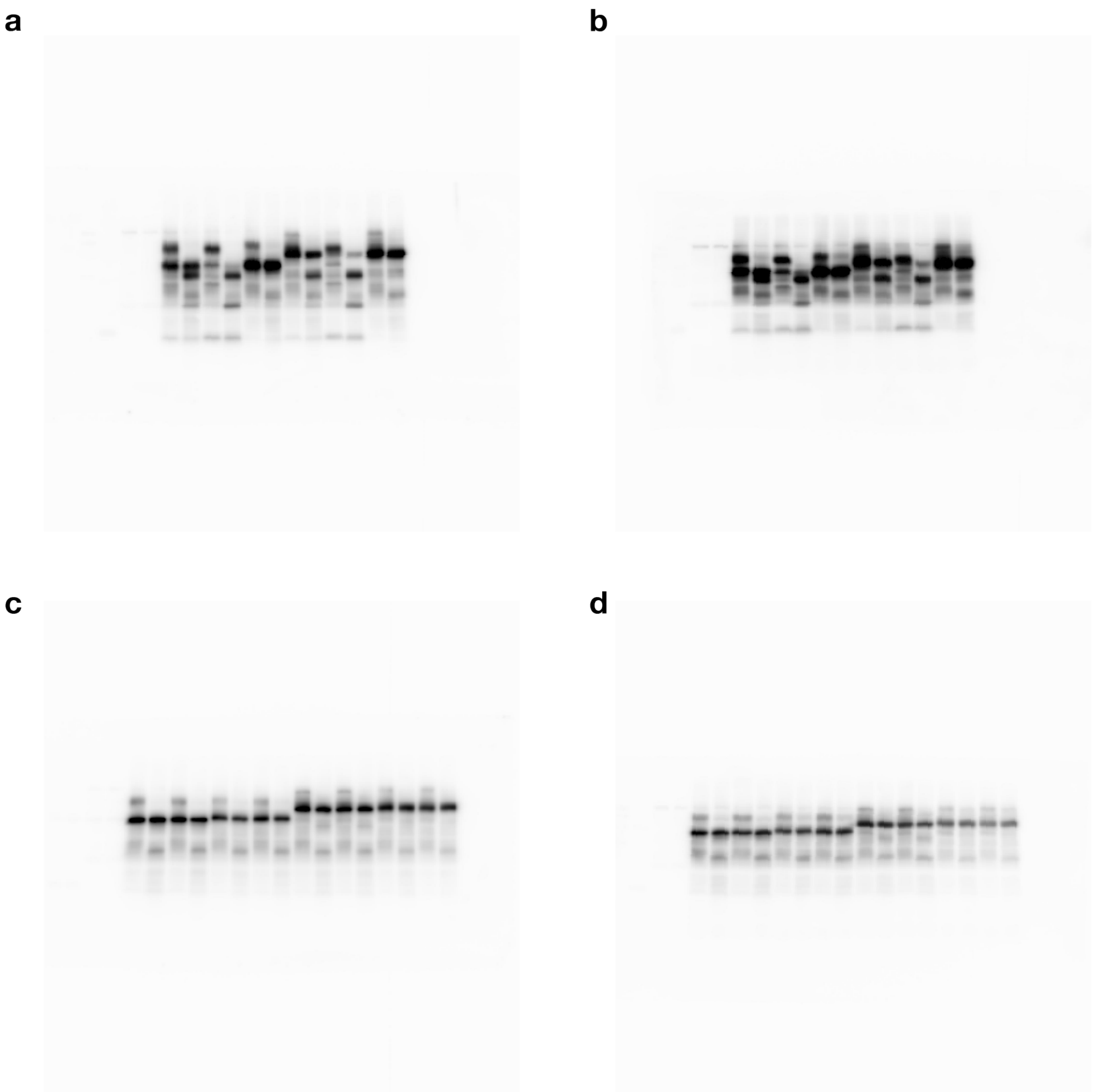

Supplementary Figure 13 Source Data for Supplementary Figure 6. (**a**) Uncropped scans of the Western blots shown in Figure 6a, including (**b**) additional scan of uncropped image from the second independent experiment. (**c**) Uncropped scans of the Western blots shown in Figure 6a, including (**d**) additional scan of uncropped image from the second independent experiment.

Supplementary Table 1: *B. subtilis* strains

strains	genotype	parent	plasmid	ref
PY79	*wildtype*			7
SCB2619	*rplWΩkan*			8
SCB2634	*rplW(d65-69)Ωkan*			8
SCB2656	*rpsSΩkan*			8
SCB2917	*rpsSΩkanΩrplV(d86-90)*			8
SCB2942	*rplD(d66-70)Ωkan*			8
SCB3349	*rpsSΩkanΩrplV(Ec-loop)*			8
SCB3958	*amyE::*$_{PmifM}$ *gfp-apcA(62-108)-FLAG-lacZΩcat*	PY79	pCH2124	9
SCB3959	*amyE::*$_{PmifM}$ *gfp-apdA(39-128)-FLAG-lacZΩcat*	PY79	pCH2125	9
SCB3999	*amyE::*$_{PmifM}$ *gfp-apdA(39-128)(R120A)-FLAG-lacZΩcat*	PY79	pCH2134	9
SCB4349	*rplWΩkan, amyE::*$_{PmifM}$ *gfp-myc-lacZΩcat*	SCB2619	pCH2311	*
SCB4352	*rplWΩkan, amyE::*$_{PmifM}$ *gfp-apdA(39-128)-myc-lacZΩcat*	SCB2619	pCH2318	*
SCB4353	*rplWΩkan, amyE::*$_{PmifM}$ *gfp-apdP(34-140)-myc-lacZΩcat*	SCB2619	pCH2320	*
SCB4354	*rplWΩkan, amyE::*$_{PmifM}$ *gfp-mifM(35-95)-myc-lacZΩcat*	SCB2619	pCH2323	*
SCB4356	*rplW(d65-69)Ωkan, amyE::*$_{PmifM}$ *gfp-myc-lacZΩcat*	SCB2634	pCH2311	*
SCB4359	*rplW(d65-69)Ωkan, amyE::*$_{PmifM}$ *gfp-apdA(39-128)-myc-lacZΩcat*	SCB2634	pCH2318	*
SCB4360	*rplW(d65-69)Ωkan, amyE::*$_{PmifM}$ *gfp-apdP(34-140)-myc-lacZΩcat*	SCB2634	pCH2320	*
SCB4361	*rplW(d65-69)Ωkan, amyE::*$_{PmifM}$ *gfp-mifM(35-95)-myc-lacZΩcat*	SCB2634	pCH2323	*
SCB4363	*rplD(d66-70)Ωkan, amyE::*$_{PmifM}$ *gfp-myc-lacZΩcat*	SCB2942	pCH2311	*
SCB4366	*rplD(d66-70)Ωkan, amyE::*$_{PmifM}$ *gfp-apdA(39-128)-myc-lacZΩcat*	SCB2942	pCH2318	*
SCB4367	*rplD(d66-70)Ωkan, amyE::*$_{PmifM}$ *gfp-apdP(34-140)-myc-lacZΩcat*	SCB2942	pCH2320	*
SCB4368	*rplD(d66-70)Ωkan, amyE::*$_{PmifM}$ *gfp-mifM(35-95)-myc-lacZΩcat*	SCB2942	pCH2323	*
SCB4370	*rpsSΩkan, amyE::*$_{PmifM}$ *gfp-myc-lacZΩcat*	SCB2656	pCH2311	*
SCB4373	*rpsSΩkan, amyE::*$_{PmifM}$ *gfp-apdA(39-128)-myc-lacZΩcat*	SCB2656	pCH2318	*
SCB4374	*rpsSΩkan, amyE::*$_{PmifM}$ *gfp-apdP(34-140)-myc-lacZΩcat*	SCB2656	pCH2320	*
SCB4375	*rpsSΩkan, amyE::*$_{PmifM}$ *gfp-mifM(35-95)-myc-lacZΩcat*	SCB2656	pCH2323	*
SCB4377	*rpsSΩkanWrplV(d86-90), amyE::*$_{PmifM}$ *gfp-myc-lacZΩcat*	SCB2917	pCH2311	*
SCB4380	*rpsSΩkanWrplV(d86-90), amyE::*$_{PmifM}$ *gfp-apdA(39-128)-myc-lacZΩcat*	SCB2917	pCH2318	*
SCB4381	*rpsSΩkanWrplV(d86-90), amyE::*$_{PmifM}$ *gfp-apdP(34-140)-myc-lacZΩcat*	SCB2917	pCH2320	*
SCB4382	*rpsSΩkanWrplV(d86-90), amyE::*$_{PmifM}$ *gfp-mifM(35-95)-myc-lacZΩcat*	SCB2917	pCH2323	*
SCB4392	*rpsSΩkanWrplV(Ec-loop), amyE::*$_{PmifM}$ *gfp-myc-lacZΩcat*	SCB3349	pCH2311	*
SCB4396	*rpsSΩkanWrplV(Ec-loop), amyE::*$_{PmifM}$ *gfp-apdA(39-128)-myc-lacZΩcat*	SCB3349	pCH2318	*
SCB4397	*rpsSΩkanWrplV(Ec-loop), amyE::*$_{PmifM}$ *gfp-apdP(34-140)-myc-lacZΩcat*	SCB3349	pCH2320	*
SCB4398	*rpsSΩkanWrplV(Ec-loop), amyE::*$_{PmifM}$ *gfp-mifM(35-95)-myc-lacZΩcat*	SCB3349	pCH2323	*

*This study

Supplementary Table 2: Plasmids

plasmid	gene	ref	PCR 1			PCR 2		
			fw primer 1	rv primer 1	template 1	fw primer 2	rv primer 2	template 2
pCH2125	gfp-apdA(39-128)-FLAG-lacZ	[9]						
pCH2126	gfp-apdP(34-140)-FLAG-lacZ	(1)						
pCH2128	gfp-apdP(34-140)-R131A-FLAG-lacZ	(1)						
pCH2134	gfp-apdA(39-128)-R120A-FLAG-lacZ	(1)						
pCH2311	gfp-myc-lacZ	*	myc-lacZ7-fw	GFP238-myc-rv	pSK69			
pCH2318	gfp-apdA(39-128)-myc-lacZ	*	gfp238-fw	Aj_Da-myc27-rv	pCH2125	myc-lacZ7-fw	GFP238-rv	pSK69
pCH2319	gfp-apdA(39-128)R120A-myc-lacZ	*	gfp238-fw	Aj_Da-myc27-rv	pCH2134	myc-lacZ7-fw	GFP238-rv	pSK69
pCH2320	gfp-apdP(34-140)-myc-lacZ	*	gfp238-fw	Sm_Db-myc27-rv	pCH2126	myc-lacZ7-fw	GFP238-rv	pSK69
pCH2321	gfp-apdP(34-140)R131A-myc-lacZ	*	gfp238-fw	Sm_Db-myc27-rv	pCH2128	myc-lacZ7-fw	GFP238-rv	pSK69
pCH2323	gfp-mifM(35-95)-myc-lacZ	*	gfp238-mifM35-fw2	mifM-myc27-rv	PY79 chrDNA	myc-lacZ7-fw	GFP238-rv	pSK69
pCH2427	gfp-apdA(39-128)-myc-lacZ-CTC	*	myc-lacZ7-CTC-fw	myc27-rv	pCH2318			
pCH2428	gfp-apdA(39-128)R120A-myc-lacZ-CTC	*	myc-lacZ7-CTC-fw	myc27-rv	pCH2319			
pCH2429	gfp-apdP(34-140)-myc-lacZ-CTC	*	myc-lacZ7-CTC-fw	myc27-rv	pCH2320			
pCH2430	gfp-apdP(34-140)R131A-myc-lacZ-CTC	*	myc-lacZ7-CTC-fw	myc27-rv	pCH2321			
pCH2503	gfp-apdP(34-130)-apdA(120-128)-myc-lacZ	*	Dp130-Da120-fw	ampR 121-128(Tm62)-antisense	pCH2318	ampR 121-128(Tm62)	Dp130-rv	pCH2320
pCH2504	gfp-apdP(34-125)-apdA(115-128)-myc-lacZ	*	Dp125-Da115-fw	ampR 121-128(Tm62)-antisense	pCH2318	ampR 121-128(Tm62)	Dp125-rv	pCH2320
pCH2505	gfp-apdP(34-120)-apdA(110-128)-myc-lacZ	*	Dp120-Da110-fw	ampR 121-128(Tm62)-antisense	pCH2318	ampR 121-128(Tm62)	Dp120-rv	pCH2320
pCH2506	gfp-apdP(34-115)-apdA(105-128)-myc-lacZ	*	Dp115-Da105-fw	ampR 121-128(Tm62)-antisense	pCH2318	ampR 121-128(Tm62)	Dp115-rv	pCH2320
pCH2507	gfp-apdA(39-119)-apdP(131-140)-myc-lacZ	*	Da119-Dp131-fw	ampR 121-128(Tm62)-antisense	pCH2320	ampR 121-128(Tm62)	Da119-rv	pCH2318
pCH2508	gfp-apdA(39-114)-apdP(126-140)-myc-lacZ	*	Da114-Dp126-fw	ampR 121-128(Tm62)-antisense	pCH2320	ampR 121-128(Tm62)	Da114-rv	pCH2318
pCH2509	gfp-apdA(39-109)-apdP(121-140)-myc-lacZ	*	Da109-Dp121-fw	ampR 121-128(Tm62)-antisense	pCH2320	ampR 121-128(Tm62)	Da109-rv	pCH2318
pCH2510	gfp-apdA(39-104)-apdP(116-140)-myc-lacZ	*	Da104-Dp116-fw	ampR 121-128(Tm62)-antisense	pCH2320	ampR 121-128(Tm62)	Da104-rv	pCH2318
pHuXBP1	Human XBP1u(186-261)	(1)						
pKIG1344	T7 promoter-CrPV IRES on pUC18	*	pUC vec fw	Plac Drbs rv	pUC18	Plac Drbs-T7 fw	MifM5GS pUC rv	GeneArt
pKIG1354	CrPV-3xFLAG-gfp-apdA(39-128)-myc-lacZ179	*	pUC vec fw	MifM5gs-3F rv	pKIG1344	3f-lGFP fw	lacZ 179-pUC1 rv	pCH2427
pKIG1355	CrPV-3xFLAG-gfp-apdA(39-128)R120A-myc-lacZ179	*	pUC vec fw	MifM5gs-3F rv	pKIG1344	3f-lGFP fw	lacZ 179-pUC1 rv	pCH2428
pKIG1356	CrPV-3xFLAG-gfp-apdP(34-140)-myc-lacZ179	*	pUC vec fw	MifM5gs-3F rv	pKIG1344	3f-lGFP fw	lacZ 179-pUC1 rv	pCH2429
pKIG1357	CrPV-3xFLAG-gfp-apdP(34-140)R131A-myc-lacZ179	*	pUC vec fw	MifM5gs-3F rv	pKIG1344	3f-lGFP fw	lacZ 179-pUC1 rv	pCH2430
pKIG1387	CrPV-3xFLAG-gfp-XBP1u(186-261)-myc-lacZ179	*	myc24 fw	GFP238-rv	pKIG1354	GFP-XBP1u fw	XBP1u-myc rv2	pHuXBP1
pKIG1388	CrPV-3xFLAG-gfp-XBP1u(186-261)S255A-myc-lacZ179	*	myc24 fw	GFP238-rv	pKIG1354	GFP-XBP1u fw	XBP1u_S255A-myc rv	pHuXBP1
pKIG1389	CrPV-3xFLAG-gfp-XBP1u(186-261)W256A-myc-lacZ179	*	myc24 fw	GFP238-rv	pKIG1354	GFP-XBP1u fw	XBP1u_W256A-myc rv	pHuXBP1
pUC18	cloning vector	(2)						

(1) Gift from Dr. Satoshi Naito

(2) TaKaRa

* This study

Supplementary Table 3: Primers

Primer name	sequence (5' to 3')
3f-lGFP fw	GATTATAAAGATCATGACATCGATTACAAGGATGACGATGACAAGAAGCTTACTAGTAGTAAAGGAGAA
Aj_Da-myc27-rv	ATCCTCTTCTGAGATGAGTTTTTGTTCGACGGCTACCGGGAAAGGAGG
ampR 121-128(Tm62)	GCAGTGCTGCCATAACCATGAGTG
ampR 121-128(Tm62)-antisense	CACTCATGGTTATGGCAGCACTGC
Da104-Dp116-fw	AGCGAGCTCCACGACGAAAAAACCAGAATTGCGCGTCTC
Da104-rv	TTCGTCGTGGAGCTCGCTGAG
Da109-Dp121-fw	GAAGCAGCTCCCGCCTCGCGTCTCCCATCCTGTCAGAGC
Da109-rv	CGAGGCGGGAGCTGCTTCGTC
Da114-Dp126-fw	TCGCGCACGGCGAACCGCCAGAGCAAGTGCATTCGCGCG
Da114-rv	GCGGTTCGCCGTGCGCGAGGC
Da119-Dp131-fw	CGCCTCGCGCTGGGTGATCGCGCGCCGCCAGCGGCGGGA
Da119-rv	ATCACCCAGCGCGAGGCGGTT
Dp115-Da105-fw	CCTTCCCTGCCGGTGGCGGCAGCTCCCGCCTCGCGCACG
Dp115-rv	CGCCACCGGCAGGGAAGGAGC
Dp120-Da110-fw	GCGAAAACCAGAATTGCGCGCACGGCGAACCGCCTCGCG
Dp120-rv	CGCAATTCTGGTTTTCGCCAC
Dp125-Da115-fw	GCGCGTCTCCCATCCTGTCTCGCGCTGGGTGATCGCGCT
Dp125-rv	ACAGGATGGGAGACGCGCAAT
Dp130-Da120-fw	TGTCAGAGCAAGTGCATTCGCGCTCCTCCTTTCCCGGTA
Dp130-rv	AATGCACTTGCTCTGACAGGA
GFP-XBP1u fw	GGCATGGATGAACTATACAAAATCTCCCCATGGATTCTGGC
gfp238-fw	GGCATGGATGAACTATACAAA
gfp238-mifM35-fw2	GGCATGGATGAACTATACAAACGGGAGTGCCGTGCGGGCGAG
GFP238-myc-rv	ATCCTCTTCTGAGATGAGTTTTTGTTCTTTGTATAGTTCATCCATGCC
GFP238-rv	TTTGTATAGTTCATCCATGCC
lacZ 179-pUC1 rv	GGGTGTCGGGGCTGGCTTAATTATTAGCGTAAAAATGCGCTCAGGTC
lacZ120-TAATAA-21rv	TGGTGCCGGAAACCAGGCAAATTATTACGGATTCTCCGTGGGAACAAA
lacZ60-TAATAA-21-rv	TGGTGCCGGAAACCAGGCAAATTATTAGCGCCATTCGCCATTCAGGCT
mifM-myc27-rv	ATCCTCTTCTGAGATGAGTTTTTGTTCTAAAAGAAGAGAACCGGCGTC
MifM5GS pUC rv	GGGTGTCGGGGCTGGCTTAATTGGATCCCACAAACATTGT
MifM5gs-3F rv	ATCGATGTCATGATCTTTATAATCACCGTCATGGTCTTTGTAGTCGGATCCCACAAACATTGTCAT
myc-lacZ7-CTC-fw	AAACTCATCTCAGAAGAGGATCTCTCACTCGCCGTCGTTTTACAA
myc-lacZ7-fw	AAACTCATCTCAGAAGAGGATCTGTCACTGGCCGTCGTTTTACAA
myc24 fw	GAACAAAAACTCATCTCAGAAGAG
myc27-rv	ATCCTCTTCTGAGATGAGTTTTTGTTC
Plac Drbs rv	TGTGTGAAATTGTTATCCGC
Plac Drbs-T7 fw	GCGGATAACAATTTCACACAGGGCCTAATACGACTCACTATA
PT7 fw2	GGGCCTAATACGACTCACTA
PT7-RBSkf-GFP	TAACTTTAAGAAGGAGGGAGATATACCAATGACAATGTTTGTGGGATC
pUC vec fw	TTAAGCCAGCCCCGACACCC
Sm_Db-myc27-rv	ATCCTCTTCTGAGATGAGTTTTTGTTCAAGGAAGGCTCCCGCCGCTGG
Universal-primer-77(PURE)	GAAATTAATACGACTCACTATAGGGAGACCACAACGGTTTCCCTCTAGAAATAATTTTGTTTAACTTTAA GAAGGAG
XBP1u_S255A-myc rv	CTCTTCTGAGATGAGTTTTTGTTCGTTCATTAATGGCTTCCATGCTGGCTGATGACGTCC
XBP1u_W256A-myc rv	CTCTTCTGAGATGAGTTTTTGTTCGTTCATTAATGGCTTTGCGGATGGCTGATGACGTCC
XBP1u-myc rv2	CTCTTCTGAGATGAGTTTTTGTTCGTTCATTAATGGCTTCCAGGA

Supplementary Table 4: Preparation of template DNA for *in vitro* translation

gene	1st PCR			2nd PCR*		system
	fw primer 1	rv primer 1	template 1	fw primer 2	rv primer 2	
gfp-apdA-lacZa	PT7-RBSkf-GFP	lacZ60-TAATAA-21-rv	pCH2318	Universal-primer-77(PURE)	lacZ60-TAATAA-21-rv	PUREfrex
gfp-apdP-lacZa	PT7-RBSkf-GFP	lacZ60-TAATAA-21-rv	pCH2320	Universal-primer-77(PURE)	lacZ60-TAATAA-21-rv	PUREfrex
gfp-apdP130-A120-lacZa	PT7-RBSkf-GFP	lacZ60-TAATAA-21-rv	pCH2503	Universal-primer-77(PURE)	lacZ60-TAATAA-21-rv	PUREfrex
gfp-apdP125-A115-lacZa	PT7-RBSkf-GFP	lacZ60-TAATAA-21-rv	pCH2504	Universal-primer-77(PURE)	lacZ60-TAATAA-21-rv	PUREfrex
gfp-apdP120-A110-lacZa	PT7-RBSkf-GFP	lacZ60-TAATAA-21-rv	pCH2505	Universal-primer-77(PURE)	lacZ60-TAATAA-21-rv	PUREfrex
gfp-apdP115-A105-lacZa	PT7-RBSkf-GFP	lacZ60-TAATAA-21-rv	pCH2506	Universal-primer-77(PURE)	lacZ60-TAATAA-21-rv	PUREfrex
gfp-apdA119-P131-lacZa	PT7-RBSkf-GFP	lacZ60-TAATAA-21-rv	pCH2507	Universal-primer-77(PURE)	lacZ60-TAATAA-21-rv	PUREfrex
gfp-apdA114-P126-lacZa	PT7-RBSkf-GFP	lacZ60-TAATAA-21-rv	pCH2508	Universal-primer-77(PURE)	lacZ60-TAATAA-21-rv	PUREfrex
gfp-apdA109-P121-lacZa	PT7-RBSkf-GFP	lacZ60-TAATAA-21-rv	pCH2509	Universal-primer-77(PURE)	lacZ60-TAATAA-21-rv	PUREfrex
gfp-apdA104-P116-lacZa	PT7-RBSkf-GFP	lacZ60-TAATAA-21-rv	pCH2510	Universal-primer-77(PURE)	lacZ60-TAATAA-21-rv	PUREfrex
3xFLAG-gfp-apdA-lacZ60	PT7 fw2	lacZ60-TAATAA-21rv	pKIG1354	–	–	RRL
3xFLAG-gfp-apdA(R120A)-lacZ60	PT7 fw2	lacZ60-TAATAA-21rv	pKIG1355	–	–	RRL
3xFLAG-gfp-apdP-lacZ60	PT7 fw2	lacZ60-TAATAA-21rv	pKIG1356	–	–	RRL
3xFLAG-gfp-apdP(R131A)-lacZ60	PT7 fw2	lacZ60-TAATAA-21rv	pKIG1357	–	–	RRL
3xFLAG-gfp-apdA-lacZ120	PT7 fw2	lacZ120-TAATAA-21rv	pKIG1354	–	–	RRL
3xFLAG-gfp-apdA(R120A)-lacZ120	PT7 fw2	lacZ120-TAATAA-21rv	pKIG1355	–	–	RRL
3xFLAG-gfp-apdP-lacZ120	PT7 fw2	lacZ120-TAATAA-21rv	pKIG1356	–	–	RRL
3xFLAG-gfp-apdP(R131A)-lacZ120	PT7 fw2	lacZ120-TAATAA-21rv	pKIG1357	–	–	RRL
3xFLAG-gfp-XBP1u-lacZ60	PT7 fw2	lacZ60-TAATAA-21rv	pKIG1387	–	–	RRL
3xFLAG-gfp-XBP1u(S255A)-lacZ60	PT7 fw2	lacZ60-TAATAA-21rv	pKIG1388	–	–	RRL
3xFLAG-gfp-XBP1u(W256A)-lacZ60	PT7 fw2	lacZ60-TAATAA-21rv	pKIG1389	–	–	RRL
3xFLAG-gfp-XBP1u-lacZ120	PT7 fw2	lacZ120-TAATAA-21rv	pKIG1387	–	–	RRL
3xFLAG-gfp-XBP1u(S255A)-lacZ120	PT7 fw2	lacZ120-TAATAA-21rv	pKIG1388	–	–	RRL
3xFLAG-gfp-XBP1u(W256A)-lacZ120	PT7 fw2	lacZ120-TAATAA-21rv	pKIG1389	–	–	RRL

* The 1st PCR product was used as the template for the 2nd PCR

Supplementary Table 5: Molecular Dynamics simulation setup of Apdp variants

Variant	Box dimensions (nm)	Total number of atoms	Number of water molecules
wild type, ↖Pro134	13.17840 13.17840 9.31854 0.00000 0.00000 0.00000 0.00000 6.58920 6.58920	200660	44125
wild type, ↙Pro134	13.17840 13.17840 9.31854 0.00000 0.00000 0.00000 0.00000 6.58920 6.58920	200660	44125
wild type, ↖↙Pro$^+$134	13.17840 13.17840 9.31854 0.00000 0.00000 0.00000 0.00000 6.58920 6.58920	200664	44126
R131A	13.17840 13.17840 9.31854 0.00000 0.00000 0.00000 0.00000 6.58920 6.58920	200657	44127
A132S	13.17840 13.17840 9.31854 0.00000 0.00000 0.00000 0.00000 6.58920 6.58920	200655	44124
P133A	13.17840 13.17840 9.31854 0.00000 0.00000 0.00000 0.00000 6.58920 6.58920	200654	44124
P134A	13.17840 13.17840 9.31854 0.00000 0.00000 0.00000 0.00000 6.58920 6.58920	200664	44126
K128A	13.17840 13.17840 9.31854 0.00000 0.00000 0.00000 0.00000 6.58920 6.58920	200665	44129
wild type, no A-site tRNA	13.17840 13.17840 9.31854 0.00000 0.00000 0.00000 0.00000 6.58920 6.58920	201031	44314

Supplementary References

1. Li W*, et al.* Structural basis for selective stalling of human ribosome nascent chain complexes by a drug-like molecule. *Nat Struct Mol Biol* **26**, 501-509 (2019).

2. Polikanov YS, Steitz TA, Innis CA. A proton wire to couple aminoacyl-tRNA accommodation and peptide-bond formation on the ribosome. *Nat Struct Mol Biol* **21**, 787-793 (2014).

3. Syroegin EA, Aleksandrova EV, Polikanov YS. Insights into the ribosome function from the structures of non-arrested ribosome-nascent chain complexes. *Nat Chem* **15**, 143-153 (2023).

4. Zhang J, Pan X, Yan K, Sun S, Gao N, Sui S-F. Mechanisms of ribosome stalling by SecM at multiple elongation steps. *eLife* **4**, (2015).

5. Su T*, et al.* The force-sensing peptide VemP employs extreme compaction and secondary structure formation to induce ribosomal stalling. *eLife* **6**, (2017).

6. Arenz S*, et al.* A combined cryo-EM and molecular dynamics approach reveals the mechanism of ErmBL-mediated translation arrest. *Nature communications* **7**, 12026 (2016).

7. Youngman P, Perkins JB, Losick R. A novel method for the rapid cloning in Escherichia coli of Bacillus subtilis chromosomal DNA adjacent to Tn917 insertions. *Mol Gen Genet* **195**, 424-433 (1984).

8. Sohmen D*, et al.* Structure of the Bacillus subtilis 70S ribosome reveals the basis for species-specific stalling. *Nat Commun* **6**, 6941 (2015).

9. Sakiyama K, Shimokawa-Chiba N, Fujiwara K, Chiba S. Search for translation arrest peptides encoded upstream of genes for components of protein localization pathways. *Nucleic Acids Res* **49**, 1550-1566 (2021).
