# Supplementary material for: RAPP-containing arrest peptides induce translational stalling by short circuiting the ribosomal peptidyltransferase activity
Source: Nat Commun. 2024 Mar 19;15:2432. doi: 10.1038/s41467-024-46761-3 (PMC10951233; doi:10.1038/s41467-024-46761-3)
Supplement: Supplementary file 3 — Description of Additional Supplementary Files [file 41467_2024_46761_MOESM3_ESM.pdf]

Description of Additional Supplementary Files

File name: Supplementary Movie 1
Description: Cryo-EM map density and model for the ApdA nascent chain. P-site tRNA (blue) with ApdA nascent chain (orange) and A-site tRNA (green) with Pro moiety (purple). Residues of RAPP motif indicated at end of rotation.

File name: Supplementary Movie 2
Description: Cryo-EM map density and model for the ApdP nascent chain. P-site tRNA (blue) with ApdP nascent chain (red) and A-site tRNA (green) with Pro moiety (cyan). Residues of RAPP motif indicated at end of rotation.
